# Restless legs syndrome in hemodialysis and peritoneal dialysis: a propensity score-matched comparison

**DOI:** 10.3389/fneur.2026.1895986

**Published:** 2026-08-19

**Authors:** Nan Hu, Tao Yuan, Lanbo Teng, Chuhan Xu, Wenyu Zhang, Wenxiu Chang

**Affiliations:** 1 Department of Nephrology, Tianjin First Central Hospital, Tianjin, China; 2 Tianjin Medical University, Tianjin, China; 3 First Teaching Hospital of Tianjin University of Traditional Chinese Medicine, Tianjin, China

**Keywords:** hemodialysis, peritoneal dialysis, Pittsburgh Sleep Quality Index, propensity score matching, restless legs syndrome, sleep disturbance, uremic toxins, β2-microglobulin

## Abstract

**Background:**

Restless legs syndrome (RLS) is highly prevalent among end-stage renal disease (ESRD) patients undergoing dialysis, causing significant sleep disruption. Whether dialysis modality influences RLS occurrence remains controversial. This study compared RLS prevalence, severity, and sleep outcomes between hemodialysis (HD) and peritoneal dialysis (PD) patients using propensity score matching.

**Methods:**

From April to October 2024, 396 ESRD patients (173 HD, 223 PD) were enrolled. RLS was diagnosed using IRLSSG 2014 criteria. Sleep quality was assessed using Pittsburgh Sleep Quality Index (PSQI ≥11 indicating clinically significant sleep disturbance). 1:1 propensity score matching was performed on age, sex, education, dialysis vintage, smoking, alcohol consumption, and comorbidities. Matched groups (140 HD, 140 PD) were compared for RLS prevalence, severity, and sleep outcomes.

**Results:**

After matching, RLS prevalence was comparable between HD and PD (39.28% vs. 40.00%, *p* = 1.0000). However, sleep disturbance was significantly more prevalent among PD patients with RLS (73.2% vs. 50.9%, *p* = 0.022). HD patients showed a trend toward more severe RLS (32.73% severe vs. 19.64% in PD, *p* = 0.754). Modality-specific risk factors, confirmed by formal interaction testing, were limited to lower hemoglobin and elevated eosinophil counts in PD patients; associations with age (HD) and alcohol consumption (PD) showed only borderline effect modification. Elevated β2-microglobulin was a shared independent risk factor with comparable effect sizes (P_interaction = 0.335).

**Conclusion:**

HD and PD showed similar RLS prevalence but divergent sleep disturbance patterns. Distinct risk factor profiles suggest dialysis-specific mechanisms differentially influence RLS pathophysiology. β2-Microglobulin emerged as a universal risk factor, supporting unified, risk factor-based screening strategies to improve sleep quality in dialysis populations regardless of modality.

## 1 Introduction

Restless legs syndrome (RLS) is a chronic sensorimotor disorder characterized by an irresistible urge to move the legs, typically accompanied by uncomfortable sensations that worsen during rest and improve with movement (1). The condition follows a circadian pattern, with symptom exacerbation in the evening and night, leading to severe sleep disruption, markedly reduced sleep quality, daytime somnolence, and significant impairment of quality of life (2–4). The sleep disturbances associated with RLS are particularly debilitating, characterized by prolonged sleep latency, frequent nocturnal awakenings, and non-restorative sleep, ultimately contributing to cardiovascular morbidity, depression, and increased mortality (3, 4). In general populations, RLS has also been associated with cognitive impairment, although this relationship has not been systematically evaluated in dialysis cohorts.

Among end-stage renal disease (ESRD) patients, RLS represents one of the most prevalent and burdensome sleep-related complications, with reported prevalence of 20–65%-substantially exceeding the 5–15% observed in the general population (5–7). Importantly, RLS is frequently underdiagnosed in dialysis populations, and its impact on sleep quality is often attributed solely to other factors such as uremic symptoms or dialysis schedules, leading to inadequate management of this treatable condition. The presence of RLS in ESRD patients is independently associated with worse sleep quality, increased cardiovascular events, and higher mortality risk, underscoring the critical need for systematic screening and targeted intervention (6, 7).

The pathophysiology of RLS in ESRD is multifactorial, involving uremic toxin accumulation, iron deficiency, dopaminergic dysfunction, peripheral neuropathy, and chronic inflammation (8, 9). Both hemodialysis (HD) and peritoneal dialysis (PD) may differentially influence RLS risk through their distinct clearance mechanisms—intermittent extracorporeal purification versus continuous solute exchange-with potential implications for uremic toxin accumulation and sleep continuity (10, 11).

Whether dialysis modality affects RLS occurrence remains controversial, with studies reporting higher prevalence in HD (12), comparable rates (13, 14), or PD predominance [15]-discrepancies likely reflecting inadequate control for confounding rather than true modality effects (15). Furthermore, previous studies have inadequately characterized the differential impact of dialysis modality on sleep quality outcomes in RLS patients, limiting their clinical utility for sleep medicine practitioners.

Propensity score matching (PSM) offers a rigorous solution to this methodological challenge. By creating balanced pseudopopulations based on measured confounders, PSM approximates randomized trial conditions, enabling more valid causal inference (16).

This study aimed to: (1) compare RLS prevalence, severity, and sleep quality outcomes between HD and PD patients after rigorous propensity score matching; (2) identify modality-specific and shared risk factors for RLS and sleep disturbance; and (3) elucidate whether observed associations reflect dialysis-specific mechanisms or underlying uremic pathophysiology, with the ultimate goal of informing evidence-based screening strategies to improve sleep quality in dialysis populations.

## 2 Materials and methods

### 2.1 Study design and setting

This single-center, cross-sectional observational study was conducted at the Department of Nephrology, Tianjin First Central Hospital, a tertiary academic medical center in China, from April to October 2024. Patients were enrolled consecutively upon presentation to the dialysis unit or outpatient clinic during the study period, minimizing selection bias. The study protocol was approved by the Institutional Ethics Committee (approval number: [2023DZX52]) and conducted in accordance with the Declaration of Helsinki. All participants provided written informed consent.

### 2.2 Participants

Consecutive ESRD patients undergoing maintenance dialysis were screened for eligibility. Inclusion criteria were: (1) age ≥18 years; (2) dialysis duration ≥3 months; (3) ability to complete structured interviews and sleep assessments. Exclusion criteria: (1) pre-existing RLS diagnosis prior to dialysis initiation; (2) Parkinson’s disease, multiple system atrophy, or other movement disorders; (3) severe cognitive impairment precluding reliable symptom reporting; (4) peripheral neuropathy from non-uremic causes (diabetes without uremia, vitamin B12 deficiency, alcohol-related); (5) lower limb amputation, severe arthritis, or orthopedic surgery; (6) concurrent sleep disorders including obstructive sleep apnea, periodic limb movement disorder, narcolepsy, or circadian rhythm disorders; (7) acute infection, active malignancy, or unstable medical condition; (8) incomplete data precluding analysis.

### 2.3 Sample size considerations

Patients were enrolled consecutively during the study period; therefore, no formal *a priori* sample size calculation was performed. *Post hoc* adequacy assessment indicated that the matched sample (140 patients per group) provided approximately 92% power to detect a 20-percentage-point difference in RLS prevalence (40% vs. 60%; two-sided alpha = 0.05), and 80% power to detect differences of ~16 percentage points - magnitudes smaller than those reported in previous uncontrolled comparisons (13, 17). The 95% confidence interval for the observed prevalence difference (−10.5 to 12.0%) excluded clinically meaningful between-modality differences. For the sleep disturbance comparison among RLS patients (55 vs. 56), the study had 80% power to detect differences of ~26 percentage points. Multivariable models achieved events-per-variable ratios of 13.8 (HD) and 14.0 (PD), exceeding the recommended minimum of 10 (18).

### 2.4 Dialysis protocols

HD patients received thrice-weekly sessions (4 h each) using polysulfone dialyzers with bicarbonate-buffered dialysate. PD patients performed continuous ambulatory PD (CAPD) or automated PD (APD) with glucose-based dialysate (1.5, 2.5%, or 4.25% dextrose). All patients received standard ESRD care including erythropoiesis-stimulating agents, phosphate binders, and active vitamin D as indicated.

### 2.5 RLS and sleep assessment

RLS diagnosis was established through structured face-to-face interviews conducted by trained nephrologists using the IRLSSG 2014 diagnostic criteria (1): (1) urge to move legs, usually with uncomfortable sensations; (2) onset/worsening during rest or inactivity; (3) partial/complete relief with movement; (4) evening/night predominance; and (5) exclusion of mimicking conditions (myalgia, venous stasis, leg edema, arthritis, cramps, positional discomfort). Severity was graded as: mild (occasional symptoms, minimal sleep disturbance); moderate (<2 episodes weekly, moderate sleep interference, mild daytime dysfunction); or severe (≥3 episodes weekly, severe sleep disruption with daytime symptoms) (1).

Sleep quality was comprehensively assessed using the Pittsburgh Sleep Quality Index (PSQI), a validated 19-item self-rated questionnaire evaluating sleep quality over the previous month (19). The PSQI generates seven component scores (subjective sleep quality, sleep latency, sleep duration, habitual sleep efficiency, sleep disturbances, use of sleeping medication, and daytime dysfunction) and a global score ranging from 0 to 21. While the traditional cutoff of >5 distinguishes poor from good sleepers in general populations (19), we adopted a global PSQI score ≥11 to identify clinically significant sleep disturbance requiring further clinical attention. This threshold has been increasingly validated in chronic disease populations: in Parkinson’s disease, PSQI scores of 11–21 were classified as significant sleep problems warranting clinical attention, in contrast to milder difficulties (scores 5–10) (20); and in nursing populations, scores of 11–15 reflect fair sleep quality indicating clinically relevant sleep compromise (21). We considered this cutoff appropriate for our dialysis population, where patients with PSQI ≥11 may benefit from more intensive clinical evaluation and targeted intervention to improve sleep outcomes (19, 22). Sleep assessments were conducted on non-dialysis days for HD patients and during routine clinic visits for PD patients to standardize evaluation conditions.

RLS severity was graded during the same structured interview according to a three-level classification integrating symptom frequency with its impact on sleep and daytime function, with thresholds anchored in the clinical course specifiers of the IRLSSG 2014 consensus criteria (intermittent, <2 episodes/week; chronic-persistent, ≥2 episodes/week) (1) and the DSM-5 frequency criterion (≥3 episodes/week) (23), and dimensions mirroring those of the International RLS Study Group rating scale (symptom frequency, sleep disturbance, and daytime functioning) (24): mild (occasional episodes with minimal interference with sleep onset and no significant distress); moderate (no more than two episodes per week, with definite difficulty initiating sleep, moderate sleep interference, and mild daytime functional impairment); or severe (three or more episodes per week, with severe disruption of nocturnal sleep and overt daytime symptoms).

### 2.6 Data collection

Demographic, clinical, and laboratory data were extracted from electronic medical records and standardized interviews. Variables included: age, sex, education level (≤primary, middle, high school, ≥college), dialysis modality and vintage, comorbidities (hypertension, diabetes mellitus, coronary heart disease [CHD], cerebrovascular disease), lifestyle factors (smoking, alcohol consumption), and medications. Laboratory parameters were obtained from routine monthly assessments: complete blood count with differential, comprehensive metabolic panel, iron studies (serum iron, ferritin), parathyroid hormone (PTH), and β2-microglobulin. Medication data were extracted for iron supplementation, phosphate binders, and sedative/hypnotic use. However, detailed information on specific RLS-targeted medications (dopamine agonists, gabapentinoids, pregabalin), antidepressants (particularly SSRIs and SNRIs, which may exacerbate RLS symptoms), and antihistamines was not systematically collected, as these medications are not routinely prescribed in our dialysis unit and their use was not formally documented in the electronic medical records.

### 2.7 Propensity score matching

To address baseline imbalances between HD and PD groups, we performed 1:1 nearest-neighbor PSM with caliper width of 0.08 (25). The propensity score-probability of receiving HD versus PD-was estimated using multivariable logistic regression with the following covariates selected based on prior evidence of associations with both dialysis modality and RLS: age, sex, education level, dialysis vintage, smoking, alcohol consumption, hypertension, diabetes mellitus, CHD, and cerebrovascular disease. Matching balance was assessed using standardized mean differences (SMD), with |SMD| < 0.10 indicating adequate balance.

### 2.8 Statistical analysis

Matched HD and PD groups were compared for: (1) RLS prevalence using chi-square test; (2) RLS severity using Cochran–Mantel–Haenszel test for ordinal data; (3) sleep quality parameters (PSQI global and component scores) using independent t-tests or Mann–Whitney U tests; and (4) continuous characteristics using independent t-tests or Mann–Whitney U tests as appropriate. Within each modality, RLS cases and non-cases were compared using the above tests. Variables with *p* < 0.10 in univariate analysis were entered into multivariable logistic regression models to identify independent risk factors. Given collinearity between hemoglobin, red blood cell count, and hematocrit (r > 0.85), only hemoglobin was retained. Effect sizes were expressed as odds ratios (OR) with 95% confidence intervals (CI). Correlation between RLS severity and PSQI scores was assessed using Spearman’s rank correlation. Between-modality comparisons accounted for the matched-pair design: binary outcomes (RLS prevalence, sleep disturbance) were compared using McNemar’s exact test, and ordinal/continuous outcomes (RLS severity scored 0–3, PSQI scores) using the Wilcoxon signed-rank test, with pair membership taken from the matching matrix. Effect modification by dialysis modality was evaluated using pooled logistic regression models including multiplicative modality × risk factor interaction terms, adjusted for the propensity score covariates; two-sided P_interaction <0.05 was considered evidence of effect modification (26).

Primary between-modality contrasts were limited to three pre-specified outcomes: RLS prevalence, severity distribution, and sleep disturbance prevalence. Within-modality comparisons in Table 1 were considered exploratory; univariate *p* values were used solely to screen candidate variables (*p* < 0.10) for multivariable models. To address multiplicity, Benjamini–Hochberg false discovery rate (FDR) correction was applied *post hoc* across Table 1 comparisons within each modality, with adjusted q < 0.05 considered significant.

**Table 1:** Comparison of clinical characteristics between RLS and non-RLS patients in hemodialysis (HD) and peritoneal dialysis (PD) groups.

Characteristic	Hemodialysis (HD)			Peritoneal dialysis (PD)		
	non-RLS (*n* = 85)	RLS (*n* = 55)	*p* value	non-RLS (*n* = 84)	RLS (*n* = 56)	*p* value
Sex, male (%)	54 (63.5)	34 (61.8)	0.980	52 (61.9)	35 (62.5)	1.000
Age, years	51.92 ± 12.14	58.11 ± 13.02	0.006**	53.50 (42.75, 62.25)	57.5 (47.50, 64.25)	0.228
Dialysis vintage, months	24 (11, 72)	60 (16, 117)	0.009**	36 (18, 55.5)	33 (17.5, 72)	0.430
Education ≤middle school, *n* (%)	59(69.4)	41 (74.5)	0.226	61(72.7)	43 (76.8)	0.924
Hypertension, Yes (%)	81 (95.3)	53 (96.4)	1.000	77 (91.7)	55 (98.2)	0.145
Diabetes mellitus, Yes (%)	24 (28.2)	21 (38.2)	0.296	23 (27.4)	23 (41.1)	0.132
Coronary heart disease, Yes (%)	22 (25.9)	24 (43.6)	0.046*	22 (26.2)	23 (41.1)	0.097
Cerebrovascular disease, Yes (%)	14 (16.5)	11 (20.0)	0.759	11 (13.1)	12 (21.4)	0.284
Smoking, Yes (%)	10 (11.8)	10 (18.2)	0.417	10 (11.9)	10 (17.9)	0.460
Alcohol consumption, Yes (%)	4 (4.7)	3 (5.5)	1.000	2 (2.4)	7 (12.5)	0.030*
Iron supplementation, Yes (%)	45 (52.9)	25 (45.5)	0.489	55 (65.5)	40 (71.4)	0.580
Phosphate binders, Yes (%)	9 (10.6)	2 (3.6)	0.201	58 (69.0)	35 (62.5)	0.535
CRP (mg/L)	5.66 (1.93, 14.91)	5.57 (2.14, 11.69)	0.881	3.59 (1.94, 7.37)	3.46 (2.44, 6.13)	0.764
Prealbumin (mg/dL)	30.70 ± 7.84	29.31 ± 7.35	0.288	31.30 (27.04, 37.45)	32.39 (27.54, 38.32)	0.372
Albumin (g/L)	40.30 (37.70, 42.60)	39.50 (37.35, 42.15)	0.345	37.4 (34.50, 40.00)	37.65 (34.98, 39.95)	0.845
LDL-C (mmol/L)	2.00 (1.55, 2.63)	1.92 (1.49, 2.62)	0.613	2.20 (1.66, 2.99)	2.20 (1.64, 3.03)	0.843
HDL-C (mmol/L)	0.78 (0.64, 0.95)	0.84 (0.63, 1.08)	0.449	0.90 (0.77, 1.05)	0.94 (0.74, 1.13)	0.623
Hemoglobin (g/L)	113.01 ± 14.73	110.49 ± 11.59	0.261	114.50 (107.75, 118.50)	104.0 (97.75, 112.50)	<0.001***
WBC (×10^9^/L)	6.47 (5.50, 7.77)	6.12 (5.17, 7.28)	0.355	7.18 (5.59, 8.56)	6.54 (5.79, 8.00)	0.623
Platelet (×10^9^/L)	175 (143, 219)	179 (142.50, 226.50)	0.815	230 (174.25, 261.25)	217.50 (166.25, 264.50)	0.827
BUN (mmol/L)	25.13 (20.55, 28.51)	24.87 (20.87, 29.43)	0.801	18.83 ± 5.53	19.99 ± 4.92	0.194
Creatinine (μmol/L)	987.1 (769.7, 1174.7)	1001.4 (840.85, 1160.05)	0.525	954.25 (736.33, 1188.08)	990.75 (797.30, 1165.95)	0.625
Uric acid (μmol/L)	408.24 ± 96.63	412.03 ± 78.95	0.800	352.4 (308.53, 399.95)	335.95 (278.68, 377.25)	0.110
Calcium (mmol/L)	2.21 (2.09, 2.32)	2.19 (2.10, 2.33)	0.959	2.25 (2.15, 2.35)	2.22 (2.11, 2.32)	0.206
Phosphorus (mmol/L)	2.01 ± 0.59	1.96 ± 0.67	0.641	1.61 (1.40, 1.79)	1.69 (1.43, 1.90)	0.251
Potassium (mmol/L)	4.87 ± 0.67	4.97 ± 0.73	0.381	4.25 (3.75, 4.81)	4.21 (3.93, 4.81)	0.985
Sodium (mmol/L)	138.1 (136.5, 140.1)	138.2 (135.8, 140.2)	0.942	140.4 (138.6, 142.13)	140.4 (138.4, 142.03)	0.804
CO₂ (mmol/L)	19.8 (18.4, 22.0)	20.4 (18.64, 21.75)	0.958	25.86 ± 3.28	25.83 ± 2.83	0.945
ALP (U/L)	71.90 (58.40, 92.50)	87.90 (69.15, 106.35)	0.003**	93.15 (69.05, 119.85)	86.65 (72.33, 124.15)	0.818
Total cholesterol (mmol/L)	3.85 (3.16, 4.81)	3.60 (2.99, 4.49)	0.401	4.08 (3.41, 5.09)	4.35 (3.64, 5.05)	0.680
Triglycerides (mmol/L)	1.90 (1.28, 3.21)	1.59 (0.89, 2.61)	0.179	1.58 (1.13, 2.22)	1.52 (0.97, 2.20)	0.790
PTH (pg/mL)	238.8 (131.4, 369.9)	289.0 (167.65, 344.70)	0.401	286.30 (149.35, 498.80)	276.05 (165.43, 444.80)	0.975
Serum iron (μmol/L)	11.8 (9.2, 15.7)	10.7 (8.85, 15.6)	0.302	13.45 (9.78, 16.48)	13.25 (9.10, 17.48)	0.893
Ferritin (ng/mL)	147 (87.3, 269.0)	140 (100.65, 206.0)	0.585	91.55 (64.73, 153.25)	114.00 (59.75, 170.00)	0.605
β₂-MG (mg/L)	27.40 ± 8.17	34.13 ± 5.77	<0.001***	22.82 ± 6.98	29.45 ± 8.96	<0.001***
Neutrophils (×10^9^/L)	4.77 (3.75, 6.12)	4.42 (3.59, 5.22)	0.288	4.65 (3.51, 5.78)	4.36 (3.62, 6.01)	0.925
Monocytes (×10^6^/L)	380 (300, 490)	370 (305, 460)	0.809	440 (358, 565)	410 (335, 558)	0.354
Eosinophils (×10^9^/L)	0.170 (0.090, 0.300)	0.170 (0.095, 0.300)	0.964	0.165 (0.110, 0.220)	0.260 (0.188, 0.333)	<0.001***
Basophils (×10^6^/L)	30 (20, 40)	30 (20, 40)	0.826	30 (20, 40)	30 (30, 50)	0.127
CRP/Albumin ratio	0.1429 (0.047, 0.394)	0.1359 (0.052, 0.324)	0.908	0.101 (0.051, 0.187)	0.095 (0.062, 0.170)	0.833
Sleeping pills, Yes (%)	23 (27.1)	14 (25.5)	0.989	4 (4.8)	5 (8.9)	0.484
Sleep disturbance, Yes (%)	24 (28.2)	28 (50.9)	0.011*	27 (32.1)	41 (73.2)	<0.001***
PSQI score	6 (4, 11)	11 (9, 16)	<0.001***	7 (5, 12)	12 (9, 15)	<0.001***

Data are presented as mean ± SD, median (IQR), or *n* (%).

RLS, restless legs syndrome; CRP, C-reactive protein; LDL-C, low-density lipoprotein cholesterol; HDL-C, high-density lipoprotein cholesterol; WBC, white blood cell; BUN, blood urea nitrogen; PTH, parathyroid hormone; β₂-MG, β₂-microglobulin; ALP, alkaline phosphatase.

*p* values were calculated using Student’s t-test, Mann–Whitney U test, or Chi-square test as appropriate.

*p < 0.05, **p < 0.01, ***p < 0.001.

Among RLS patients specifically, PD patients had significantly worse PSQI sleep latency scores (2.0 [1.0, 3.0] vs. 1.0 [0.0, 2.0], *p* = 0.018) and sleep disturbances scores (2.0 [1.0, 2.0] vs. 1.0 [0.0, 2.0], *p* = 0.031) compared to HD patients.

For risk factors significant in both modalities, we tested for effect size heterogeneity using the Cochran Q statistic and I^2^ index. A significant Q statistic (*p* < 0.10) or I^2^ > 50% suggested meaningful between-modality differences. For sensitivity analyses, we performed: (1) inverse probability of treatment weighting (IPTW) using propensity scores as alternative balancing method; (2) stratified analysis by dialysis vintage (<24 vs. ≥24 months); and (3) exclusion of APD patients to assess CAPD-specific effects. All analyses used R software (version 4.4.2) with packages MatchIt, survey, meta, and rms. Two-tailed *p* < 0.05 indicated statistical significance.

## 3 Results

### 3.1 Enrollment and grouping

**Figure 1:**
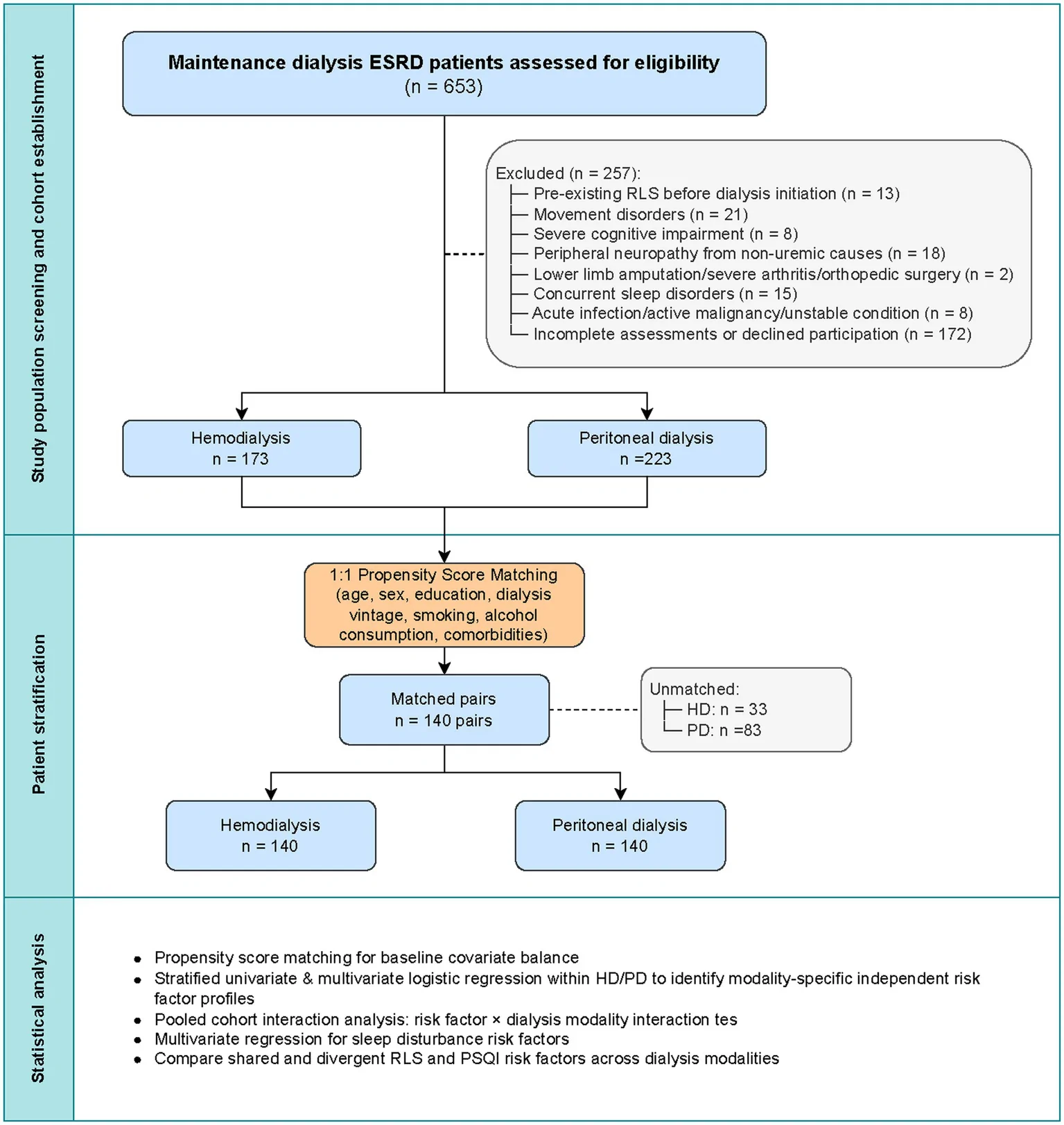
Participant flow diagram, including screening, eligibility exclusions with reasons, enrollment, 1:1 propensity score matching, and analysis populations. HD, Hemodialysis; PD, Peritoneal dialysis; PSM, Propensity score matching; IPTW, Inverse probability of treatment weighting.

Between April and October 2024, 653 consecutive ESRD patients undergoing maintenance dialysis at our center were assessed for eligibility. Of these, 257 were excluded for pre-specified reasons: pre-existing RLS diagnosis prior to dialysis initiation (*n* = 13), movement disorders (*n* = 21), severe cognitive impairment (*n* = 8), peripheral neuropathy from non-uremic causes (*n* = 18), lower limb amputation or orthopedic conditions (*n* = 2), concurrent sleep disorders (*n* = 15), acute infection, active malignancy, or unstable medical condition (*n* = 8), and incomplete assessments or declined participation (*n* = 172). The remaining 396 eligible patients were enrolled and completed all study assessments, including structured face-to-face interviews, RLS evaluation, and the PSQI: 173 receiving hemodialysis and 223 receiving peritoneal dialysis (Figure 1).

### 3.2 Baseline characteristics before and after matching

**Figure 2:**
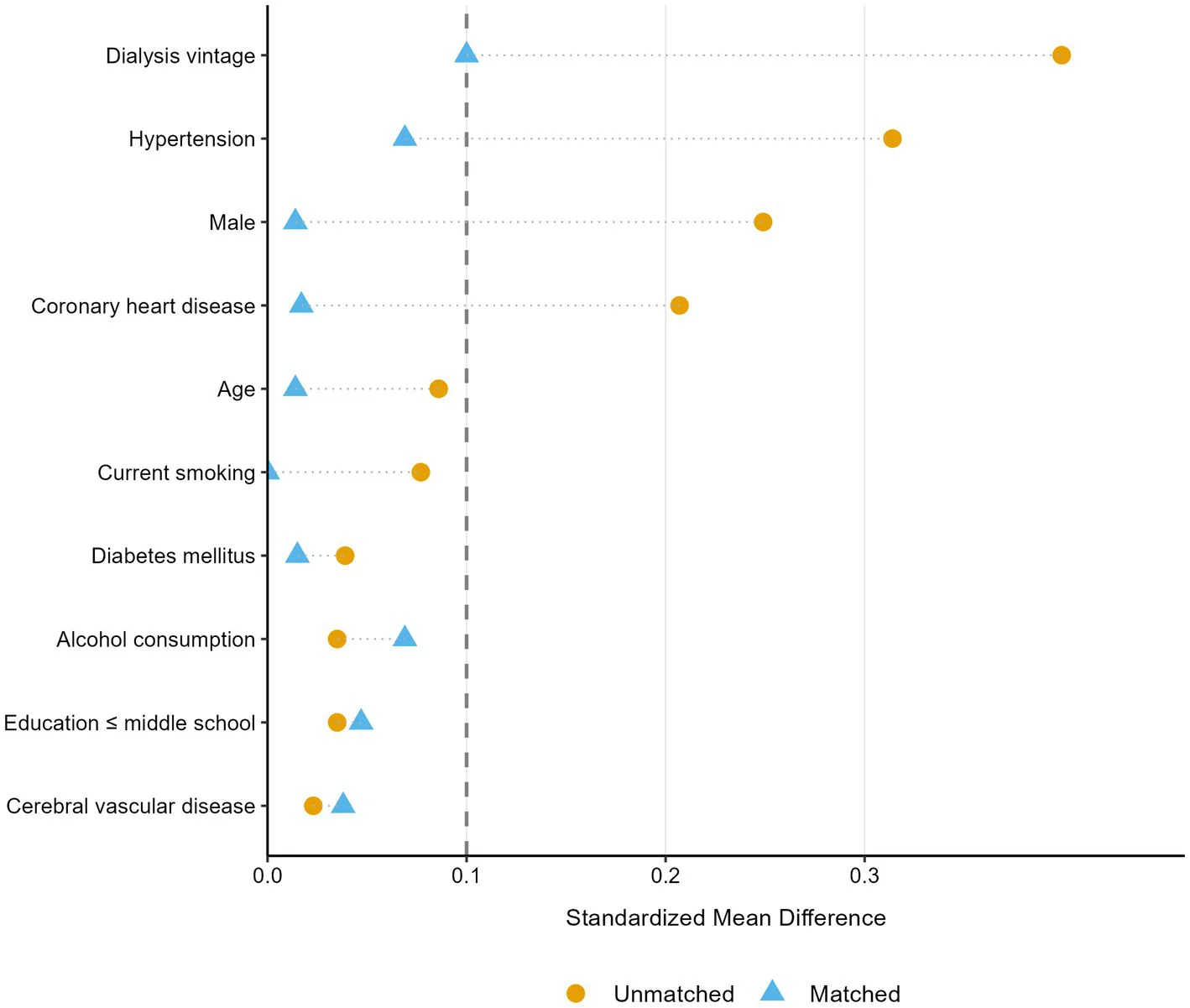
Standardized mean differences before and after propensity score matching. Dashed lines indicate |SMD| = 0.10 threshold for adequate balance.

Of 396 enrolled patients, 173 received HD and 223 PD. Before matching, significant baseline differences existed: HD patients were more likely male (65.3% vs. 52.9%), had longer dialysis vintage (43 [14–107] vs. 30 [16–60] months), and higher hypertension prevalence (89.0% vs. 95.5%) (Table 2, left columns). After 1:1 PSM, 140 matched pairs were analyzed. All standardized mean differences were <0.1, indicating excellent covariate balance (Table 2, right columns; Figure 2).

**Table 2:** Baseline characteristics before and after propensity score matching.

Characteristic	Before matching			After matching		
	HD (*n* = 173)	PD (*n* = 223)	*p* value	HD (*n* = 140)	PD (*n* = 140)	*p* value
Demographics						
Age, years	55 (45–64)	54 (44–62)	0.469	54.5 (44.8–64.0)	55.0 (44.0–64.0)	0.906
Male sex, *n* (%)	113 (65.3)	118 (52.9)	0.017	88 (62.9)	87 (62.1)	1.000
Education ≤middle school, *n* (%)	119 (68.8)	157 (70.4)	0.813	100 (71.4)	104 (74.3)	0.687
Dialysis characteristics						
Dialysis vintage, months	43 (14–107)	30 (16–60)	0.010	36 (14–96)	36 (18–60)	0.336
Comorbidities, *n* (%)						
Hypertension	154 (89.0)	213 (95.5)	0.023	134 (95.7)	132 (94.3)	0.784
Diabetes mellitus	59 (34.1)	72 (32.3)	0.785	45 (32.1)	46 (32.9)	1.000
Coronary heart disease	59 (34.1)	56 (25.1)	0.065	46 (32.9)	45 (32.1)	1.000
Cerebrovascular disease	31 (17.9)	38 (17.0)	0.924	25 (17.9)	23 (16.4)	0.874
Lifestyle factors, *n* (%)						
Current smoking	27 (15.6)	29 (13.0)	0.554	20 (14.3)	20 (14.3)	1.000
Alcohol consumption	9 (5.2)	10 (4.5)	0.924	7 (5.0)	9 (6.4)	0.797

HD, hemodialysis; PD, peritoneal dialysis; SMD, standardized mean difference.

### 3.3 RLS prevalence, severity, and sleep outcomes

In the matched cohort, RLS prevalence was nearly identical: 39.28% (55/140) in HD versus 40.00% (56/140) in PD (absolute difference 0.72, 95% CI: −10.5 to 12.0%; McNemar exact *p* = 1.000, 33 vs. 34 discordant pairs).

**Figure 3:**
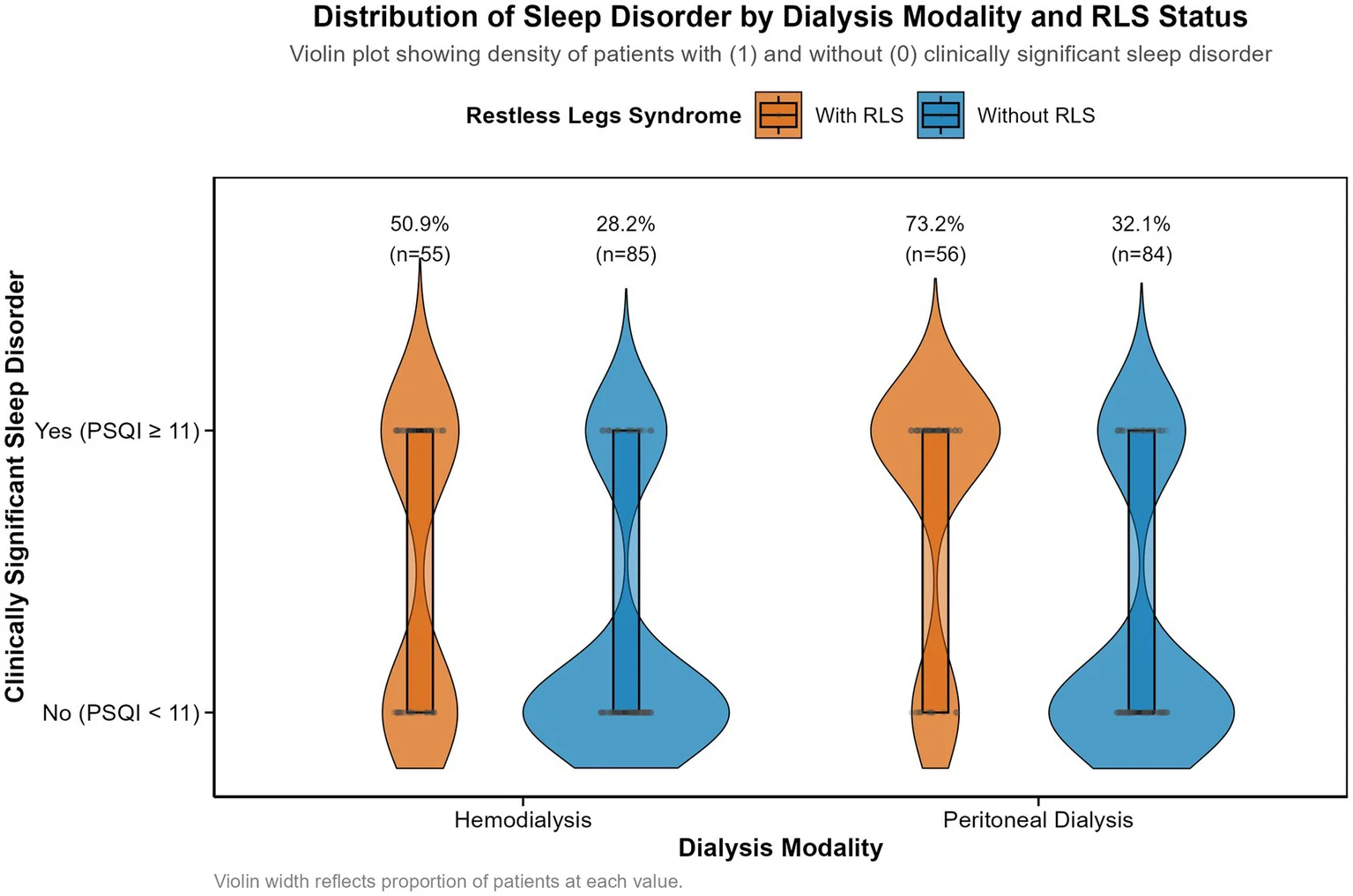
Sleep disorder distribution by dialysis modality in the propensity score-matched cohort.

Severity distribution showed a non-significant trend toward greater severity in HD (paired Wilcoxon signed-rank test on pair-level severity scores, *p* = 0.754): mild HD 50.91% (28/55), PD 51.79% (29/56); moderate HD 16.36% (9/55), PD 28.57% (16/56); severe HD 32.73% (18/55), PD 19.64% (11/56) (Figure 3).

Critically, sleep disturbance (PSQI ≥11) showed a striking modality-specific pattern among RLS patients: PD patients with RLS experienced significantly more sleep disturbance compared to HD patients with RLS (73.2% vs. 50.9%, *p* = 0.022). This represents a 44% higher rate of clinically significant sleep disturbance in PD patients with RLS. Among patients without RLS, sleep disturbance rates were comparable between modalities (HD 28.2% vs. PD 32.1%, *p* = 0.580), indicating that the differential sleep impact is specific to the RLS phenotype (Figure 3).

PSQI component analysis revealed that PD patients with RLS had significantly worse scores in sleep latency (*p* = 0.018) and sleep disturbances (*p* = 0.031) compared to HD patients with RLS, with trends toward worse subjective sleep quality and daytime dysfunction (Table 1).

### 3.4 Risk factor profiles by dialysis modality

Table 1 presents the detailed comparison of clinical characteristics between RLS and non-RLS patients within each dialysis modality.

In the HD group, patients with RLS were significantly older than those without RLS (58.11 ± 13.02 vs. 51.92 ± 12.14 years, *p* = 0.006). RLS patients also had markedly longer dialysis vintage (60 [16–117] vs. 24 [11–72] months, *p* = 0.009) and higher prevalence of coronary heart disease (43.6% vs. 25.9%, *p* = 0.046). Sleep disturbance was significantly more common in HD patients with RLS (50.9% vs. 28.2%, *p* = 0.011). Laboratory findings revealed that HD patients with RLS had significantly higher alkaline phosphatase levels (87.90 [69.15–106.35] vs. 71.90 [58.40–92.50] U/L, *p* = 0.003) and markedly elevated β2-microglobulin concentrations (34.13 ± 5.77 vs. 27.40 ± 8.17 mg/L, *p* < 0.001). RLS severity in HD patients showed moderate positive correlation with PSQI global scores (r = 0.42, *p* = 0.002), indicating that more severe RLS translated directly into worse sleep quality.

In the PD group, distinct risk factors emerged. Patients with RLS had significantly higher alcohol consumption rates (12.5% vs. 2.4%, *p* = 0.030) and substantially lower hemoglobin levels (104 [98–113] vs. 115 [108–119] g/L, *p* < 0.001) compared to those without RLS. Notably, eosinophil counts were significantly elevated in PD patients with RLS (0.260 [0.188–0.333] vs. 0.155 [0.110–0.220] × 10^9^/L, p < 0.001). Similar to the HD group, β2-microglobulin was significantly higher in PD patients with RLS (29.45 ± 8.96 vs. 22.82 ± 6.98 mg/L, *p* < 0.001). Sleep disturbance was also more prevalent among PD patients with RLS (73.2% vs. 32.1%, *p* < 0.001). The correlation between RLS severity and PSQI scores was stronger in PD patients (r = 0.51, *p* < 0.001), suggesting that RLS symptomatology may have a more pronounced impact on sleep architecture in this modality. In contrast to HD, age (57.5 vs. 53.5 years, *p* = 0.228), dialysis vintage (33 vs. 36 months, *p* = 0.430), and coronary heart disease prevalence (41.1% vs. 26.2%, *p* = 0.097) did not significantly differ between PD patients with and without RLS, although coronary heart disease showed a borderline association.

### 3.5 Multivariable risk factor analysis

Multivariable analysis identified age (OR 1.051, 95% CI 1.015–1.089, *p* = 0.006) and β2-microglobulin (OR 1.161, 95% CI 1.085–1.243, *p* < 0.001) as independent risk factors for RLS in HD patients. CHD showed borderline significance (OR 2.057, 95% CI 0.883–4.791, *p* = 0.095) (Table 3).

**Table 3:** Multivariable logistic regression for restless legs syndrome risk factors by dialysis modality.

Variable	Hemodialysis			P for interaction	Variable	Peritoneal Dialysis			P for interaction
	OR	95% CI	*p* value			OR	95% CI	*p* value	
Age, per year	1.051	1.015–1.089	0.006	0.086	Alcohol consumption	6.369	1.026–39.553	0.047	0.073
Dialysis vintage, per month	1.002	0.995–1.009	0.541	0.609	Hemoglobin, per g/L	0.931	0.891–0.973	0.002	0.001
Coronary heart disease	2.057	0.883–4.791	0.095	0.822	Eosinophil count, per 0.1 × 10^9^/L	1.593	1.095–2.317	0.015	<0.001
β2-microglobulin, per mg/L	1.161	1.085–1.243	<0.001	0.335	β2-microglobulin, per mg/L	1.092	1.032–1.155	0.002	0.335

OR, odds ratio; CI, confidence interval.

PD patients with RLS demonstrated distinct characteristics: higher alcohol consumption (12.5% vs. 2.4%, *p* = 0.030), lower hemoglobin (104 [98–113] vs. 115 [108–119] g/L, *p* < 0.001), and elevated eosinophil counts (0.260 [0.188–0.333] vs. 0.155 [0.110–0.220] × 10^9^/L, *p* < 0.001) (Table 1). β2-microglobulin was similarly elevated (29.45 ± 8.96 vs. 22.82 ± 6.98 mg/L, *p* < 0.001).

Multivariable analysis identified alcohol consumption (OR 6.369, 95% CI 1.026–39.553, *p* = 0.047), hemoglobin (OR 0.931, 95% CI 0.891–0.973, *p* = 0.002), β2-microglobulin (OR 1.092, 95% CI 1.032–1.155, *p* = 0.002), and eosinophil count (OR 1.593, 95% CI 1.095–2.317, *p* = 0.015) as independent risk factors for RLS in PD patients (Table 3).

Variables with *p* < 0.10 in the univariate analysis for sleep disorders were entered into the multivariate logistic regression model to determine independent risk factors. Results of the univariate analysis were not presented. Multivariate analysis showed that in hemodialysis patients, age (OR = 1.050, 95%CI: 1.016–1.087, *p* = 0.004) and β2-microglobulin (OR = 1.053, 95%CI: 1.023–1.098, *p* = 0.002) were independent risk factors for sleep disorders. In peritoneal dialysis patients, β2-microglobulin (OR = 1.062, 95%CI: 1.034–1.121, *p* < 0.001) and eosinophil count (OR = 1.647, 95%CI: 1.105–2.450, *p* = 0.008) were independent risk factors for sleep disorders. In addition, decreased hemoglobin (OR = 0.934, 95%CI: 0.883–0.983, *p* = 0.021) was also an independent risk factor for sleep disorders (Table 4). In hemodialysis patients, the independent risk factors for RLS and sleep disorders were identical. In peritoneal dialysis patients, risk factors for sleep disorders were included in those for RLS.

**Table 4:** Multivariable logistic regression for sleep disorder risk factors.

Variable	Hemodialysis			Variable	Peritoneal dialysis		
	OR	95% CI	*p* value		OR	95% CI	*p* value
Age, per year	1.050	1.016–1.087	0.004	Age, per year	1.024	0.988–1.062	0.197
β2-microglobulin, per mg/L	1.053	1.023–1.098	0.002	Phosphate binders	0.308	0.129–0.903	0.072
Phosphate binders	0.130	0.007–0.786	0.065	Hemoglobin, g/L	0.934	0.883–0.983	0.021
Prealbumin, mg/dL	0.964	0.912–1.016	0.176	β2-microglobulin, per mg/L	1.062	1.034–1.121	<0.001
CO₂, mmol/L	0.880	0.745–1.029	0.119	Eosinophil count, per 0.1 × 10^9^/L*	1.647	1.105–2.450	0.008
Ferritin, ng/mL	1.002	1.000–1.004	0.112	Diabetes mellitus	1.327	0.541–3.254	0.534
RLS	2.230	1.011–4.966	0.047	RLS	3.994	1.602–10.367	0.003

OR, odds ratio; CI, confidence interval. *Calculated as OR per 0.1 × 10^9^/L increment for interpretability.

### 3.6 Interaction testing

Interaction Testing for Modality-Specific Risk Factors. Pooled logistic regression models with modality × risk factor interaction terms (adjusted for propensity score covariates) confirmed significant effect modification for hemoglobin (P_interaction = 0.001) and eosinophil count (P_interaction<0.001), and confirmed homogeneity of the β2-microglobulin effect (P_interaction = 0.335), consistent with the Cochran Q analysis (*p* = 0.272, I^2^ = 17%). Only borderline interactions were observed for age (*p* = 0.086) and alcohol consumption (*p* = 0.073), and no effect modification was detected for dialysis vintage (*p* = 0.609) or coronary heart disease (*p* = 0.822) (Table 3).

### 3.7 Shared risk factor: β2-microglobulin and sleep outcomes

**Figure 4:**
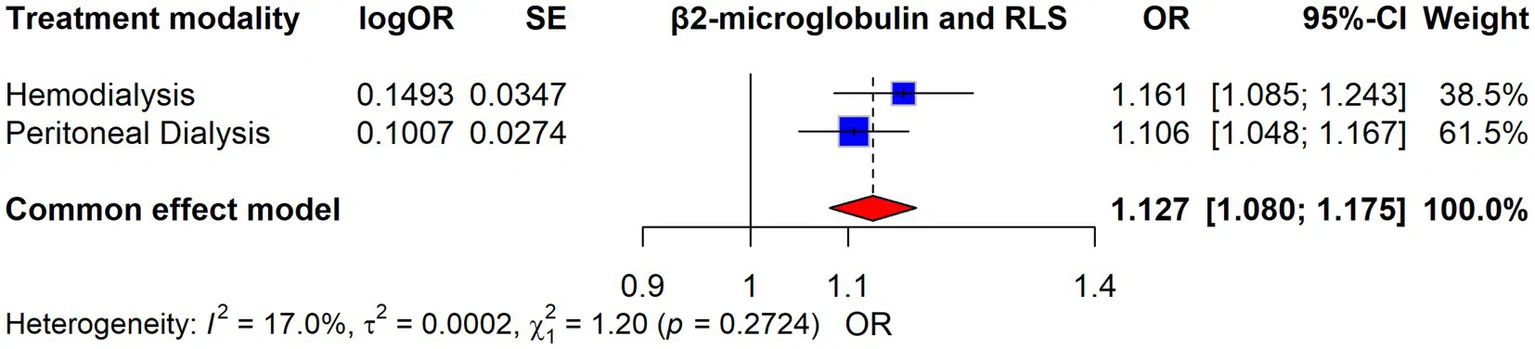
Forest plot comparing β_2_-microglobulin effect sizes between hemodialysis and peritoneal dialysis. OR, odds ratio; CI, confidence interval.

β2-Microglobulin emerged as the only independent risk factor common to both modalities. Despite higher absolute levels in HD, the effect sizes were comparable: HD OR 1.161 versus PD OR 1.092 per 1 mg/L increase. Formal comparison revealed no significant heterogeneity (Q = 1.20, p = 0.272; I^2^ = 17.0%), supporting a consistent, modality-independent association (Figure 4).

Notably, β2-microglobulin levels also showed positive correlation with PSQI scores in both HD (r = 0.38, *p* < 0.001) and PD (r = 0.44, *p* < 0.001) patients, suggesting that this uremic toxin may mediate its clinical impact partly through sleep disruption mechanisms.

### 3.8 Sensitivity analyses

IPTW analysis on the full cohort yielded nearly identical results: adjusted RLS prevalence HD 38.9% versus PD 40.2% (*p* = 0.89). Stratification by dialysis vintage showed consistent findings in both short- and long-duration subgroups. Exclusion of 23 APD patients (retaining 117 CAPD pairs) did not materially alter results (prevalence HD 39.3% vs. CAPD 39.3%, *p* = 1.00; sleep disturbance in RLS patients: HD 51.2% vs. CAPD 72.3%, *p* = 0.028).

## 4 Discussion

From a sleep medicine perspective, this rigorously designed study employing propensity score matching provides critical evidence that dialysis modality itself is not a primary determinant of RLS occurrence, but significantly influences the prevalence of associated sleep disturbance and the risk factors that drive RLS pathophysiology. We demonstrate that hemodialysis and peritoneal dialysis are associated with virtually identical RLS prevalence (~40%) and comparable severity distributions. However, PD patients with RLS experienced a significantly higher prevalence of sleep disturbance than their HD counterparts (73.2% vs. 50.9% with PSQI ≥11), despite having less severe RLS symptoms on average. Formal interaction testing confirmed PD-specific associations for anemia and eosinophilia, whereas the associations of age with RLS in HD and of alcohol consumption with RLS in PD showed only borderline effect modification and should be regarded as exploratory. Elevated β2-microglobulin represented a shared, modality-independent risk factor with comparable effect sizes, suggesting it reflects underlying uremic pathophysiology rather than dialysis-specific mechanisms.

### 4.1 RLS prevalence and dialysis modality: implications for sleep medicine practice

Our findings challenge the prevailing uncertainty regarding dialysis modality and RLS. Previous studies have yielded conflicting results: de Menezes et al. found comparable prevalence but greater severity in automated PD (13), while Al-Jahdali et al. observed higher RLS in PD (57.7% vs. 20.3%) (17). These discrepancies likely reflect methodological heterogeneity, particularly inadequate adjustment for confounding. Our PSM approach provides more robust evidence. By achieving excellent balance on key covariates (all SMD < 0.11), we minimized selection bias inherent to modality choice. The near-identical prevalence we observed suggests that previous differences likely reflected patient characteristics rather than intrinsic dialysis effects, supporting the implementation of unified RLS screening protocols across dialysis modalities in sleep medicine practice.

### 4.2 Divergent sleep outcomes: mechanistic considerations

The striking finding that PD patients with RLS experience a significantly higher prevalence of sleep disturbance despite less severe RLS symptoms warrants careful consideration from a sleep medicine standpoint. Several mechanisms may explain this paradox:

First, the continuous nature of PD may lead to more persistent nocturnal symptoms. While HD patients experience rapid solute clearance during dialysis sessions with subsequent rebound, PD provides continuous gentle clearance. This may result in more stable but persistent uremic toxin levels that maintain RLS symptoms throughout the night, leading to sleep maintenance insomnia and early morning awakenings-patterns reflected in the worse “sleep disturbances” and “sleep latency” PSQI component scores we observed in PD patients.

Second, PD-related factors may independently disrupt sleep architecture. The presence of intraperitoneal fluid, potential discomfort from the catheter, and the need for nighttime exchanges (in APD) may compound RLS-related sleep disruption. Eosinophilia, which we identified as a PD-specific risk factor, may reflect allergic or inflammatory responses to dialysate components that could independently impair sleep through cytokine-mediated mechanisms (27).

Third, the intermittent nature of HD may produce symptom fluctuations that allow for some symptom-free nights. The trend we observed toward more severe RLS in HD (32.7% vs. 19.6%) but better sleep quality suggests that when HD patients have RLS, it tends to be more severe but potentially more intermittent, whereas PD patients experience persistent, sleep-disrupting symptoms.

Recent evidence from Yakaryilmaz et al. (28) further supports the relevance of systematic symptom screening for sleep-related complications in dialysis populations. In a study of 118 older hemodialysis patients, uremic pruritus was significantly associated with worse sleep quality, greater functional dependence, and higher depression scores, with the 5-D Itch Scale score correlating positively with PSQI global scores. These findings, combined with our demonstration that RLS is a major contributor to sleep disturbance in both HD and PD patients, underscore the concept that multiple uremia-related symptoms converge to impair sleep in dialysis populations. The comprehensive geriatric assessment approach employed by Yakaryilmaz et al.-evaluating sleep, cognition, mood, nutrition, and functional status simultaneously-provides a model for future studies that seek to characterize the full symptom burden associated with RLS and other dialysis-related complications.

### 4.3 Risk factor profiles: clinical translation for sleep specialists

The age-RLS association in HD but not PD is intriguing. Age-related physiological decline-including reduced dopaminergic function, vascular stiffness, and impaired uremic toxin clearance-may synergize with HD-specific stressors (hemodynamic instability, rapid fluid shifts) to precipitate RLS (29, 30). The absence of a significant association in PD may reflect selection bias (healthier older patients choosing home therapy) or PD’s gentler physiological profile. However, the modality × age interaction was only borderline (*p* = 0.086), and the age association did not survive FDR correction; this apparent HD-specific pattern should therefore be regarded as exploratory. Given the higher absolute burden of RLS in older HD patients, prioritizing RLS screening in this group remains clinically reasonable, pending confirmation in larger cohorts.

The strong anemia-RLS association in PD contrasts with its absence in HD. This likely reflects differential erythropoietin utilization: HD patients receive more intensive ESA therapy due to thrice-weekly contact, compressing hemoglobin variability and potentially masking associations (31). The PD finding aligns with mechanistic evidence that iron deficiency and hypoxia impair dopaminergic function and exacerbate RLS (32), supporting aggressive iron optimization in PD patients presenting with sleep complaints.

Eosinophilia as a PD-specific risk factor represents a novel finding with potential sleep medicine relevance. Eosinophilic peritonitis, allergic reactions to dialysate components (particularly icodextrin), and foreign body response to the catheter may trigger systemic inflammation and immune activation relevant to RLS pathogenesis (33, 34). This observation suggests that sleep medicine practitioners evaluating PD patients with RLS should consider collaboration with nephrology teams to assess for dialysate-related reactions, particularly when standard RLS therapies fail.

Alcohol consumption emerged as a nominally significant risk factor for RLS in PD patients in both univariate and multivariable analyses. However, this association was based on very few exposed cases (7 exposed RLS cases) with a correspondingly wide confidence interval (OR 6.37, 95% CI 1.03–39.55), did not survive FDR correction, and showed only a borderline modality × alcohol interaction (P_interaction = 0.073). It should therefore be interpreted cautiously as a hypothesis-generating observation requiring confirmation in larger cohorts, rather than as established evidence of a PD-specific risk factor.

### 4.4 β2-microglobulin: a universal biomarker for sleep disruption in uremia?

β2-Microglobulin (11.8 kDa) is a well-established prototype of the “middle-molecule” uremic toxin category (molecular weight >500 Da), which is poorly cleared by conventional low-flux hemodialysis but partially removed by high-flux membranes, hemodiafiltration, and peritoneal dialysis (35–37). Its serum concentration increases progressively with declining renal function, reaching markedly elevated levels in ESRD patients. Beyond its established role as the major protein component of dialysis-related amyloidosis, accumulating evidence suggests that β2-microglobulin may exert broader neurotoxic effects relevant to RLS pathogenesis through several interconnected pathways.

#### 4.4.1 Peripheral neuropathy

Uremic peripheral neuropathy affects 60–90% of ESRD patients and is classically a distal, symmetric, sensorimotor polyneuropathy with predominant lower limb involvement (38, 39). The “middle-molecule hypothesis” of uremic neuropathy posits that neurotoxic molecules in the 300–12,000 Da range-including β2-microglobulin and parathyroid hormone-accumulate in CKD and contribute to nerve damage through axonal degeneration and demyelination (40). Given that RLS is fundamentally a sensorimotor disorder with strong peripheral nervous system involvement, the association between β2-microglobulin elevation and RLS may partially reflect shared pathogenic pathways involving peripheral nerve dysfunction (38, 39).

#### 4.4.2 Neurotoxicity

*In vitro* studies have demonstrated that β2-microglobulin oligomers exhibit direct cytotoxic effects on neuronal cells, inducing oxidative stress, mitochondrial dysfunction, and apoptosis (41). Although the blood–brain barrier (BBB) partially limits β2-microglobulin entry into the central nervous system, BBB integrity may be compromised in uremic patients due to chronic inflammation and oxidative stress (40, 41). If BBB permeability is increased, circulating β2-microglobulin could potentially affect central dopaminergic pathways and sleep–wake regulatory circuits implicated in RLS pathogenesis. This hypothesis is supported by the positive correlation we observed between β2-microglobulin levels and PSQI scores in both dialysis modalities.

#### 4.4.3 Chronic inflammation

ESRD is characterized by a state of chronic low-grade inflammation, with elevated levels of C-reactive protein, interleukin-6, and tumor necrosis factor-*α*. β2-Microglobulin itself may both reflect and amplify this inflammatory milieu: it is upregulated by inflammatory cytokines (particularly IFN-*γ* and IL-2) as a component of MHC class I antigen presentation, and its accumulation may further activate monocytes and macrophages, creating a self-perpetuating inflammatory cycle (36). Chronic inflammation has been implicated in RLS pathogenesis through multiple mechanisms, including disruption of dopaminergic neurotransmission, iron metabolism dysregulation, and peripheral nerve damage (8, 9). The independent association of β2-microglobulin with RLS in our study, observed in both dialysis modalities, may therefore reflect the integrated effect of middle-molecule retention, neurotoxicity, and chronic inflammation on sensorimotor pathways.

It should be noted that β2-microglobulin may serve as a marker of overall uremic toxin burden rather than a direct causal agent in RLS pathogenesis. The consistent association across modalities nonetheless identifies β2-microglobulin as a potentially useful biomarker for risk stratification and suggests that strategies aimed at improving middle-molecule clearance (e.g., high-flux hemodialysis, hemodiafiltration, or earlier kidney transplantation) warrant investigation as potential interventions for RLS and associated sleep disturbance in ESRD populations.

The forest plot (Figure 4) visually demonstrates the remarkable consistency of β2-microglobulin’s effect across dialysis modalities: despite substantially different absolute β2-microglobulin levels between HD and PD groups (34.13 vs. 29.45 mg/L in RLS patients), the per-unit increase in odds ratio was comparable (HD: OR 1.161; PD: OR 1.092), with no significant heterogeneity (Q = 1.20, *p* = 0.272; I^2^ = 17.0%). This finding is clinically significant because it indicates that the association between β2-microglobulin and RLS is not driven by the higher absolute levels seen in HD patients but rather reflects a dose-dependent relationship that operates across the full range of β2-microglobulin concentrations encountered in dialysis populations. The narrow and overlapping confidence intervals in Figure 3 further reinforce the precision and reliability of this cross-modality association. These data position β2-microglobulin as a potential universal risk marker for RLS in ESRD, independent of the specific dialysis technique employed, and suggest that therapeutic strategies targeting β2-microglobulin reduction could benefit patients regardless of their dialysis modality.

### 4.5 Clinical implications for sleep medicine practice

Our findings support several actionable recommendations for clinical practice:

1. Routine RLS screening in dialysis populations: given the comparable ~40% RLS prevalence across both dialysis modalities and the significant impact on sleep quality, we recommend systematic RLS screening for all maintenance dialysis patients. The IRLSSG single screening question (“In the evening or at night when you lie down, do you have unpleasant sensations in your legs?”) has demonstrated high sensitivity (85.7%) and specificity (58.3%) for RLS diagnosis in hemodialysis populations (42) and can be easily integrated into routine clinical assessments. Alternatively, the IRLS rating scale provides better specificity (81% at cutoff ≥20) and can simultaneously assess disease severity (42).
2. PSQI integration into routine dialysis assessment: the PSQI should be considered as a standard component of the dialysis patient evaluation, particularly in patients diagnosed with RLS. Our finding that 73.2% of PD patients with RLS had clinically significant sleep disturbance (PSQI ≥11) underscores that RLS screening without concurrent sleep assessment may miss the full clinical impact. The PSQI can be administered during routine clinic visits with minimal additional time burden.
3. β2-microglobulin-based risk stratification: elevated β2-microglobulin levels may serve as a practical laboratory marker to identify patients at higher risk for RLS and sleep disturbance across dialysis modalities. Given that β2-microglobulin is already routinely measured in many dialysis centers (as a marker of dialysis adequacy for middle molecules), its use as an adjunctive risk stratification tool would not require additional testing. Patients with β2-microglobulin levels above the modal-specific thresholds identified in our analysis may warrant prioritized RLS and sleep evaluation.
4. Modality-specific risk factor monitoring: in HD patients, particular attention should be paid to RLS screening in older patients with prolonged dialysis vintage and cardiovascular comorbidity. In PD patients, monitoring for anemia (hemoglobin trends), eosinophilia, and alcohol consumption patterns may help identify patients at elevated RLS risk. These parameters are already part of routine dialysis laboratory monitoring and can be incorporated into existing quality assessment frameworks.
5. Therapeutic considerations: the identification of β2-microglobulin as a shared risk factor raises the hypothesis that strategies to enhance middle-molecule clearance (e.g., high-flux dialyzers, hemodiafiltration, or adsorptive therapies) may benefit RLS symptoms and sleep quality. However, this requires confirmation through interventional trials. In the interim, evidence-based RLS treatments-including α2δ-ligands (gabapentin, pregabalin) and dopamine agonists (ropinirole, pramipexole)-should be considered for dialysis patients with clinically significant RLS, with dose adjustments for renal function (43). Iron optimization (ferritin >75 μg/L) should be ensured in all RLS patients, particularly in PD where anemia was a prominent risk factor.

### 4.6 Limitations and future directions

Our study has several limitations that warrant explicit acknowledgment. First, as a single-center, cross-sectional observational study, our findings represent associations rather than causal relationships, and the design inherently precludes causal inference and incidence assessment. The single-center design may limit generalizability to other populations with different demographic or clinical characteristics. Second, unmeasured confounding (genetic factors, residual kidney function, precise dialysate glucose exposure, objective sleep measures such as polysomnography or actigraphy) may persist despite propensity score matching. Third, the moderate sample size limits precision for subgroup analyses and may restrict the detection of modest effect sizes. Finally, while PSM effectively balanced measured confounders, it cannot address unmeasured or unobserved confounding variables that may influence both dialysis modality selection and RLS risk. Future multi-center prospective cohort studies with objective sleep monitoring and longer follow-up periods are warranted to validate our findings and establish temporal relationships. Fourth, multiple comparisons were performed; although our primary conclusions are robust to FDR correction, several secondary associations did not survive multiplicity adjustment and should be regarded as exploratory. Fifth, despite propensity score matching and concordant IPTW results, residual confounding by unmeasured factors-including residual kidney function, dialysis adequacy indices, cumulative dialysate glucose exposure, anemia-management regimens, and psychotropic or dopaminergic medications-cannot be excluded, and the reported associations are non-causal. Sixth, subgroup sizes were limited (55 HD and 56 PD RLS cases; 18 versus 11 severe cases; 23 APD patients; few exposed cases for infrequent risk factors), reducing precision for subgroup-specific estimates, as illustrated by the wide confidence interval for alcohol consumption in PD; these estimates warrant confirmation in larger multi-center cohorts. Seventh, interaction tests were based on modest numbers of outcome events (55 HD and 56 PD RLS cases) and were therefore underpowered to detect modest effect modification; borderline and non-significant interaction results should be interpreted as exploratory rather than as evidence of absent heterogeneity. Eighth, detailed medication information relevant to RLS and sleep quality-including dopamine agonists, gabapentinoids, antidepressants (particularly SSRIs/SNRIs known to potentially exacerbate RLS), and antihistamines—was not systematically collected. Although iron supplementation, phosphate binders, and sleeping pill use were recorded and did not differ significantly between RLS and non-RLS patients within each modality, residual confounding by unmeasured medications cannot be excluded. Future studies should incorporate comprehensive medication inventories, including over-the-counter medications and supplements, to more fully account for pharmacological effects on RLS and sleep outcomes. Furthermore, cognitive assessment was not performed in this study. Given that RLS has been associated with cognitive impairment in general populations, and that dialysis patients are at elevated risk for cognitive decline due to multiple shared risk factors (uremic toxin accumulation, chronic inflammation, vascular disease), the relationship between RLS and cognitive function in dialysis populations represents an important area for future investigation. Future studies should incorporate standardized cognitive assessment tools (e.g., MMSE, MoCA) alongside RLS and sleep evaluations.

Future research priorities from a sleep medicine perspective include: Multi-center prospective cohorts validating these findings and assessing RLS incidence; randomized trials comparing high-flux versus conventional HD, or biocompatible versus standard PD solutions, on objective sleep outcomes; mechanistic studies exploring β2-microglobulin’s neurotoxic pathways and effects on sleep–wake regulation; and intervention studies targeting identified risk factors with polysomnographic endpoints to improve patient outcomes.

## 5 Conclusion

In this propensity score-matched analysis of ESRD patients, hemodialysis and peritoneal dialysis demonstrated equivalent RLS prevalence but divergent sleep disturbance patterns. While HD was associated with more severe RLS symptoms, PD patients with RLS experienced significantly worse sleep quality, highlighting the need for modality-specific approaches within unified screening frameworks. Formal interaction testing confirmed PD-specific risk factors for anemia and eosinophilia, while the apparent HD- or PD-specificity of age, dialysis vintage, coronary heart disease, and alcohol consumption was not supported by significant effect modification-although the cross-sectional design cannot establish whether these represent causal, modality-specific pathways. β2-Microglobulin was the only independent risk factor consistently associated with both RLS and sleep disturbance in both HD and PD patients, suggesting it may reflect the overall burden of uremic toxin accumulation. Whether targeted reduction of β2-microglobulin can improve RLS and sleep outcomes requires confirmation through prospective interventional studies. These findings challenge dialysis modality as a primary RLS determinant and support unified, risk factor-based screening and management strategies to improve sleep quality across renal replacement therapies. Sleep medicine practitioners should prioritize systematic RLS screening in all dialysis patients, with particular attention to the distinct risk profiles and sleep outcomes associated with each modality.

## Data Availability

The original contributions presented in the study are included in the article/supplementary material, further inquiries can be directed to the corresponding authors.
